# Trajectories of Homoeolog-Specific Expression in Allotetraploid *Tragopogon castellanus* Populations of Independent Origins

**DOI:** 10.3389/fpls.2021.679047

**Published:** 2021-06-23

**Authors:** J. Lucas Boatwright, Cheng-Ting Yeh, Heng-Cheng Hu, Alfonso Susanna, Douglas E. Soltis, Pamela S. Soltis, Patrick S. Schnable, William B. Barbazuk

**Affiliations:** ^1^Advanced Plant Technology Program, Clemson University, Clemson, SC, United States; ^2^Department of Agronomy, Iowa State University, Ames, IA, United States; ^3^Covance Inc., Indianapolis, IN, United States; ^4^Botanic Institute of Barcelona, Consejo Superior de Investigaciones Científicas, ICUB, Barcelona, Spain; ^5^Department of Biology, University of Florida, Gainesville, FL, United States; ^6^Plant Molecular and Cellular Biology Program, University of Florida, Gainesville, FL, United States; ^7^Florida Museum of Natural History, University of Florida, Gainesville, FL, United States; ^8^Genetics Institute, University of Florida, Gainesville, FL, United States; ^9^Biodiversity Institute, University of Florida, Gainesville, FL, United States

**Keywords:** *Tragopogon*, allopolyploid, additive expression, homoeologs, expression bias, homoeolog-specific expression, non-model system, RNA-Seq

## Abstract

Polyploidization can have a significant ecological and evolutionary impact by providing substantially more genetic material that may result in novel phenotypes upon which selection may act. While the effects of polyploidization are broadly reviewed across the plant tree of life, the reproducibility of these effects within naturally occurring, independently formed polyploids is poorly characterized. The flowering plant genus *Tragopogon* (Asteraceae) offers a rare glimpse into the intricacies of repeated allopolyploid formation with both nascent (< 90 years old) and more ancient (mesopolyploids) formations. Neo- and mesopolyploids in *Tragopogon* have formed repeatedly and have extant diploid progenitors that facilitate the comparison of genome evolution after polyploidization across a broad span of evolutionary time. Here, we examine four independently formed lineages of the mesopolyploid *Tragopogon castellanus* for homoeolog expression changes and fractionation after polyploidization. We show that expression changes are remarkably similar among these independently formed polyploid populations with large convergence among expressed loci, moderate convergence among loci lost, and stochastic silencing. We further compare and contrast these results for *T. castellanus* with two nascent *Tragopogon* allopolyploids. While homoeolog expression bias was balanced in both nascent polyploids and *T. castellanus*, the degree of additive expression was significantly different, with the mesopolyploid populations demonstrating more non-additive expression. We suggest that gene dosage and expression noise minimization may play a prominent role in regulating gene expression patterns immediately after allopolyploidization as well as deeper into time, and these patterns are conserved across independent polyploid lineages.

## 1. Introduction

The consequences of plant polyploidization have been a subject of intense interest for several decades (reviewed in Wendel, 2000, 2015; Doyle et al., 2008; Leitch and Leitch, 2008; Van de Peer et al., 2009; Barker et al., 2012; Soltis et al., 2016). Polyploidization results in broad-scale genomic changes that serve as potentially novel avenues upon which evolution may act (reviewed in Otto and Whitton, 2000; Flagel and Wendel, 2009). Many changes occur in the generations immediately after polyploidization including changes in genome size (reviewed in Soltis et al., 2003; Leitch et al., 2008; Leitch and Leitch, 2013) spanning the extremes in both gain (e.g., *Paris japonica* Pellicer et al., 2010) and loss (e.g., *Utricularia gibba* Ibarra-Laclette et al., 2013), expression (Chen and Pikaard, 1997; reviewed in Adams and Wendel, 2005b; Chaudhary et al., 2009; Hu et al., 2015), epigenetic modifications (Shaked et al., 2001; Salmon et al., 2005; reviewed in Chen, 2007; Madlung and Wendel, 2013; Cheng et al., 2016), transposon activity (reviewed in Woodhouse et al., 2014; Vicient and Casacuberta, 2017; Wendel et al., 2018) as well as changes in protein folding and dosage (reviewed in Birchler and Veitia, 2010, 2012; Pires and Conant, 2016). These changes are variable across lineages (Anssour and Baldwin, 2010; reviewed in Soltis et al., 2016) and may occur in repeated cycles (Soltis and Soltis, 1999; Buggs et al., 2012; reviewed in Wendel, 2015; Soltis et al., 2016). In some paleopolyploids, these changes appear to largely converge over time, at least within closely related lineages (Blanc and Wolfe, 2004; reviewed in Barker et al., 2008; Edger and Pires, 2009; Freeling, 2009).

Polyploids are categorized as either autopolyploids, which are formed from a whole-genome duplication within a single species (reviewed in Otto and Whitton, 2000; Spoelhof et al., 2017), or allopolyploids, which are generated by the combination of entire genomes from two different species (Kihara and Ono, 1926). However, these definitions represent an oversimplification of the dynamic range of variability that polyploids may cover (reviewed in Stebbins, 1947; Ramsey and Schemske, 1998) and the various mechanisms by which they are formed (reviewed in Mason and Pires, 2015). Allopolyploid formation results in duplicated gene copies originating from each parent known as homoeologs. Immediately after polyploidization, homoeologs are expected to be functionally redundant and as such, one copy may be altered without deleterious effect or conserved in duplicate (reviewed in Conant et al., 2014; Pires and Conant, 2016). Whole-genome duplication in an organism can impose unfavorable dosage effects upon cellular functions unless gene balance is maintained (Freeling, 2009; Birchler and Veitia, 2010, 2012). These dosage effects likely represent one aspect of a larger framework that directs genome evolution after polyploidization (Conant et al., 2014). As such, duplicate loci in allopolyploids may experience a number of possible fates. Genomes may experience silencing or loss of one homoeologous copy via fractionation over time. Homoeolog functions may diverge from the parentally inherited state such that functions are partitioned between homoeologs (subfunctionalization), or copies may develop novel functionality (neofunctionalization) (reviewed in Edger and Pires, 2009; Freeling, 2009). Homoeologs may also interact via convergent evolution, homoeologous recombination or gene conversion (Langham et al., 2004; Doyle et al., 2008).

Expression patterns may also vary in the polyploid such that loci demonstrate spatiotemporally divergent expression from the progenitors (Pires et al., 2004b; Wang et al., 2006a; Buggs et al., 2010b; Baldauf et al., 2016), homoeolog-specific expression (HSE) (Buggs et al., 2010a; reviewed in Grover et al., 2012; Yoo et al., 2013; Woodhouse et al., 2014) or additive expression (Guo et al., 2006; Stupar and Springer, 2006; Wang et al., 2006b; reviewed in Yoo et al., 2014). HSE occurs when the polyploid expresses one parental homoeolog over the other (Woodhouse et al., 2014; Boatwright et al., 2018). HSE is similar to allele-specific expression in that both refer to expression differences that are caused by *cis*- and *trans*-regulatory variation (Bell et al., 2013), and each has been a topic of interest in hybrid and polyploid studies (Wright et al., 1998; Adams and Wendel, 2005a; Aguilar-Rangel et al., 2017). HSE differs from allele-specific expression in that HSE examines expression across homoeologous chromosomes in contrast to allele-specific expression, which examines expression between homologous chromosomes. Homoeolog expression may also diverge in an additive manner where expression in the polyploid is the arithmetic mean of the two diploid progenitors (reviewed in Yoo et al., 2014).

It is worth noting that the degree of similarity/dissimilarity in expression between parents of a polyploid and the polyploid itself, also known as parental legacy (Buggs et al., 2014), may have a significant effect upon the fate of homoeolog expression in the polyploid (Conant et al., 2014). Similarly, differences among polyploids and their diploid progenitors may derive from numerous processes such as divergent evolution of the lineages after polyploidization, effects of whole-genome duplication (i.e., larger cells and stomata, higher photosynthetic rates and gas exchange, and different stress tolerance) (Hegarty et al., 2006; Sémon and Wolfe, 2007; De Smet and Van de Peer, 2012), or hybridization (resulting in heterosis, increased genetic variation and additive expression) (Mallet, 2007; Bell et al., 2013; Soltis et al., 2016). While the fates of homoeologs after polyploidization are convergent within some lineages (Blanc and Wolfe, 2004; reviewed in Edger and Pires, 2009; and Freeling, 2009), establishing a paradigm has proved elusive (reviewed in Soltis et al., 2016).

The evolutionary model organism *Tragopogon* serves as a prominent example of repeated, naturally occurring allopolyploidization. The *Tragopogon* system includes synthetic lines, nascent (< 90 years) and meso- (~2.6 million years) polyploids (Mavrodiev et al., 2015; Soltis et al., 2016). While most species of *Tragopogon* have chromosome numbers of 2*n* = 12, there are several well-studied allopolyploids (2*n* = 24), *Tragopogon mirus, T. miscellus*, and *T. castellanus*. Both *T. mirus* and *T. miscellus* represent neoallotetraploids that formed recently in the northwestern United States after their three diploid progenitors (*T. dubius*-*T. porrifolius* and *T. dubius*-*T. pratensis*, respectively) were introduced from Europe in the early 1900s (Ownbey, 1950; Soltis et al., 2004). These allopolyploids never formed in Europe due to their geographic isolation but have formed repeatedly in the USA since the diploids were brought into close proximity. These polyploids are estimated to be approximately 45 generations old (80–90 years for these biennials) (Soltis et al., 1995; Symonds et al., 2010).

Similarly, *T. castellanus* has formed multiple times from independent allopolyploidization events (Mavrodiev et al., 2015). *Tragopogon castellanus* is endemic to Spain and occurs only along the northern half of the Iberian Peninsula (Blanca and de la Guardia, 1996). Morphological, cytological, and molecular phylogenetic analyses support *T. lamottei* and *T. crocifolius* as putative parents. *Tragopogon castellanus* is morphologically variable and somewhat similar to parental *T. crocifolius*; as a result *T. castellanus* was once considered a subspecies of *T. crocifolius* (Willkomm, 1893; de la Guardia Guerrero and López, 1989). The parentage of *T. castellanus* was validated using phylogenetic analyses of external transcribed spacers, internal transcribed spacers, *Adh*, and plastid datasets, fluorescence *in situ* hybridization, and genome *in situ* hybridization (Mavrodiev et al., 2008, 2015). *Tragopogon castellanus* may have formed before the last glacial maximum that would date the formation of this polyploid species to perhaps as long ago as 2.6 million years (Mavrodiev et al., 2015). As such, the multiple, independent occurrences of *Tragopogon* allopolyploid formation in young US species and the older *T. castellanus* permits the assessment of the fate of homoeologs in both neo- and mesopolyploids.

Previous studies have demonstrated that duplicate gene fates after polyploidization are non-random within the newly formed allopolyploid species of *Tragopogon*. That is, many gene loss and expression changes were repeated across polyploid populations of independent origins (Buggs et al., 2012; Soltis et al., 2012). However, these studies were primarily small-scale and the fates of duplicated gene copies do not generalize across all polyploid plants (reviewed in Soltis et al., 2016). Here, we examine multiple, independently formed allopolyploid *T. castellanus* lineages estimated to have formed as long as 2.6 mya (Mavrodiev et al., 2015). We show that not only are expression patterns similar, but duplicate loss is largely convergent across independent lineages of *T. castellanus*. We further compare duplicate fates in populations of this mesopolyploid from Spain to the neopolyploids from the US, *T. mirus* and *T. miscellus* (based on earlier studies; Buggs et al., 2010a,b, 2012; Boatwright et al., 2018) in which identical methods were used so that we may examine changes due to natural allopolyploidization over a large evolutionary time scale of perhaps several million years.

## 2. Materials and Methods

### 2.1. Sample Processing

Leaf tissue was collected from plants grown in controlled conditions as described by Tate et al. (2006), and RNA was extracted as described in Tate et al. (2006). Three individuals of the diploid *T. crocifolius* were sampled from the P-B lineage along with five individuals of diploid *T. lamottei* composed of two and three individuals/lineages from lineage P-I and P-II lineages, respectively (Mavrodiev et al., 2015). Both parental species are phylogenetically distinct and appeared as members of two distinct clades based on ITS phylogeny as estimated in Mavrodiev et al. (2005); namely, clade Majores s. l. [incl. clade Hebecarpus] (*T. crocifolius*) and clade Tragopogon (*T. lamottei*) (Mavrodiev et al., 2008). Sample localities and voucher information for all samples are given in Supplementary Table 1. Additional information is provided in Mavrodiev et al. (2015) and vouchers are deposited at the University of Florida herbarium (FLAS). We sequenced 12 allotetraploid *T. castellanus* individuals representing three bioreplicates for four independent polyploidization events (Supplementary Figure 1 and Supplementary Table 1). RNA-Seq samples were bar-coded and processed using the Illumina TruSeq kit.

### 2.2. Sequencing, Assembly and Annotation

Samples were sequenced using the Illumina MiSeq sequencing platform resulting in 2 X 300 paired-end reads (Supplementary Table 2). Adapters were removed using CutAdapt (Martin, 2011), and sequences were trimmed using Trimmomatic (Bolger et al., 2014). RNA reads were pooled from all individuals of each diploid species and assembled using the Trinity *de novo* assembler (Grabherr et al., 2011), resulting in one assembly per species. Redundant isoforms were removed from our assemblies using a previously described pipeline (Boatwright et al., 2018). The final assemblies were annotated using Trinotate (Altschul et al., 1990; Ashburner et al., 2000; Krogh et al., 2001; Lagesen et al., 2007; Finn et al., 2011; Grabherr et al., 2011; Kanehisa et al., 2011; Petersen et al., 2011; Powell et al., 2011; Punta et al., 2011) with default parameters (https://github.com/jlboat/Tragopogon_castellanus).

### 2.3. Ortholog Identification and Common Orthologous Regions

Putative orthologs were identified between the *T. crocifolius* and *T. lamottei* assemblies using a reciprocal best-hit approach (Moreno-Hagelsieb and Latimer, 2008) as described in Boatwright et al. (2018). Common Orthologous REgions (COREs) were identified between orthologous pairs using the local alignment provided by WU-BLAST (Gish, 2005) and a custom CPython script (https://github.com/BBarbazukLab/papers/). This resulted in BED files containing COREs that were used to filter BAM files after aligning reads to complete assemblies (Boatwright et al., 2018).

### 2.4. Poisson-Gamma Model

As in Boatwright et al. (2018), parental RNA-Seq reads were mapped to both complete, diploid references independently using Bowtie v0.12.9, -m1,-v 3] (Langmead et al., 2009) and Last [v531, -l 25] (Frith et al., 2010; Graze et al., 2012; Munger et al., 2014). The BED files containing COREs were used to filter the resulting SAM files for respective references. Parental reads that mapped uniquely within COREs were isolated, and the reads were subsequently identified as mapping equally well to both references or better to one of the two parents. A Bayesian Poisson-Gamma model (León-Novelo et al., 2014), which provides conservative estimates of the type I error (Fear et al., 2016), was used to identify COREs biasedly mapping reads from the alternative parent. COREs demonstrated expression bias if the credible interval did not overlap 0.5 for all priors (0.4, 0.5, 0.6). Polyploid reads were mapped following the same procedure, and the biased COREs—as determined by diploid read mapping—were filtered out after processing, leaving the remaining set of unbiased COREs for inference. Within the set of unbiased COREs, remaining expression bias corresponded to loci demonstrating HSE. Overlapping gene sets were visually displayed using UpSet plots generated using R (Team, 2014) and the UpSetR package (Lex et al., 2014).

### 2.5. Additively Expressed Genes

Reads mapping to both references within COREs were used to generate an expression matrix for diploids and all independent polyploids. Loci were filtered from the expression matrix that did not contain at least 10 counts-per-million based upon the average library size in 11 samples. Differentially expressed genes were identified in R (Team, 2014) using the empirical Bayesian analysis pipeline within the limma package (Ritchie et al., 2015) after using voom (Law et al., 2014) to apply precision weights to account for the mean-variance trend. Loci were considered differentially expressed at a false discovery rate of 0.05 (Benjamini and Hochberg, 1995). Contrasts were performed between *T. lamottei* and *T. crocifolius* to determine when parental expression was the same or different. To test for additivity, contrasts were performed between each population of the polyploid *T. castellanus* and its two parents where polyploid expression is expected to be the arithmetic mean of the two parental expression levels. Overlapping gene sets were visually displayed using UpSet plots generated using R (Team, 2014) and the UpSetR package (Lex et al., 2014).

### 2.6. Homoeolog Loss and Silencing

Orthologs were used to design probes for NimbleGen sequence capture to isolate genomic reads from allopolyploid *T. castellanus* individuals. Each probe was designed to target unique regions of each contig with 1-3 probes along each contig. These probes were used to isolate genomic DNA corresponding to expressed transcripts (Supplementary Table 3). Polyploid DNA reads obtained from sequence capture were aligned to diploid references in the same manner as polyploid RNA reads, and homoeolog loss and silencing were assessed within COREs using the unbiased homoeolog set. Homoeologs with mapped DNA and mapped RNA reads represent genes that are both present and expressed. Homoeologs with DNA reads and no RNA reads represent putative silencing events. Homoeologs with RNA reads but no DNA reads likely represent a failed capture or mismapped reads. Homoeologs with neither DNA nor RNA reads represent putative loss. Overlapping gene sets were visually displayed using UpSet plots generated using R (Team, 2014) and the UpSetR package (Lex et al., 2014).

### 2.7. Functional Protein Association Network and GO Enrichment

Loci common to all independently formed polyploid populations that were lost, exhibited additive or non-additive expression as well as loci that demonstrated HSE were individually tested for interaction enrichment. *Arabidopsis thaliana* orthologs were identified for *Tragopogon* contigs using WU-BLAST blastx with an *A. thaliana* protein database. The *E*-value cutoff was set at 1E-75, and the high-scoring segment pair had to represent 70% of either the total query or subject length. The resulting lists of *A. thaliana* genes were used to construct functional protein association networks using STRING10 (Szklarczyk et al., 2014). The resulting networks used only high-confidence, experimentally validated protein-protein interactions with disconnected nodes in the networks hidden, and the edge thickness represented confidence of data supporting interaction. Protein-protein interaction enrichment *p*-values were FDR corrected (Benjamini and Hochberg, 1995). All gene sets were further checked for GO enrichment using GOSeq (Young et al., 2012). The background for HSE and lost loci was the set of unbiased COREs. The background for additively expressed genes included all loci tested for additivity. Functional network details and GO annotations are available at (https://github.com/jlboat/Tragopogon_castellanus).

## 3. Results

### 3.1. Assembly, Annotation and Ortholog Identification

The assemblies of the diploids, *T. crocifolius* and *T. lamottei*, contained 113,865 and 155,600 contigs, respectively. Assemblies were annotated using Trinotate, and putative orthologs and domains were identified. For each of the diploid species, over 7,000 entries hit *Arabidopsis thaliana* sequences, and approximately 500 of the remainder hit *Oryza sativa* ssp. *japonica* using NCBI-BLAST against the SwissProt database. We also identified approximately 4,800 unique eggNOG hits for each diploid, where eggNOG hits represent hierarchical orthologous groups and provide functional annotations for homologous sequences. We identified 14,388 orthologs between the diploid genomes and delimited COREs for downstream processing (Gish, 2005; Moreno-Hagelsieb and Latimer, 2008). COREs were assessed for similarity in both length and GC content (Supplementary Figures 1, 2) and were found to be highly similar between the two species, with length differences never exceeding 16 bases and GC content differences primarily falling under 2%.

### 3.2. Additive Expression

**Figure 1:**
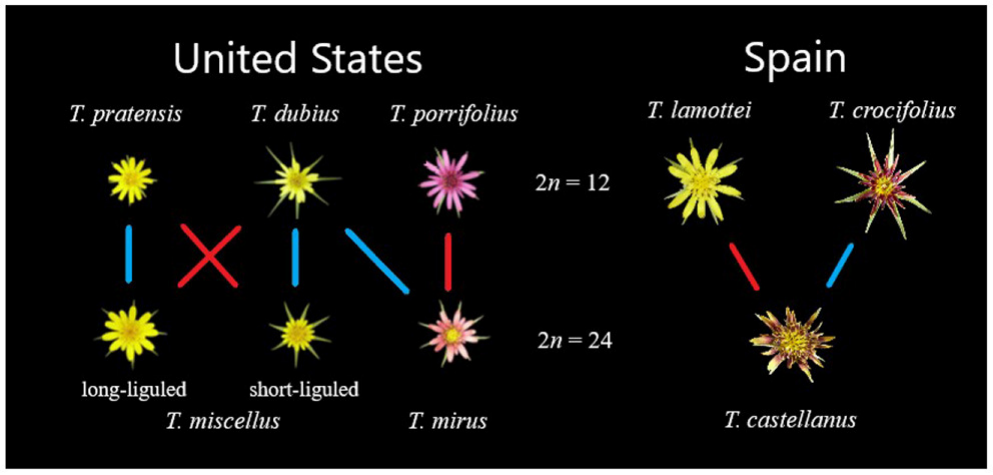
Relationships among the *Tragopogon* diploids and polyploids. US species are left of the chromosome counts and Spanish species are on the right. Diploids are aligned along the top row and polyploid offspring are along the bottom row. Colored lines indicate whether the diploid serves as the maternal or paternal parent for the corresponding polyploid, where blue is paternal and red is maternal.

**Figure 2:**
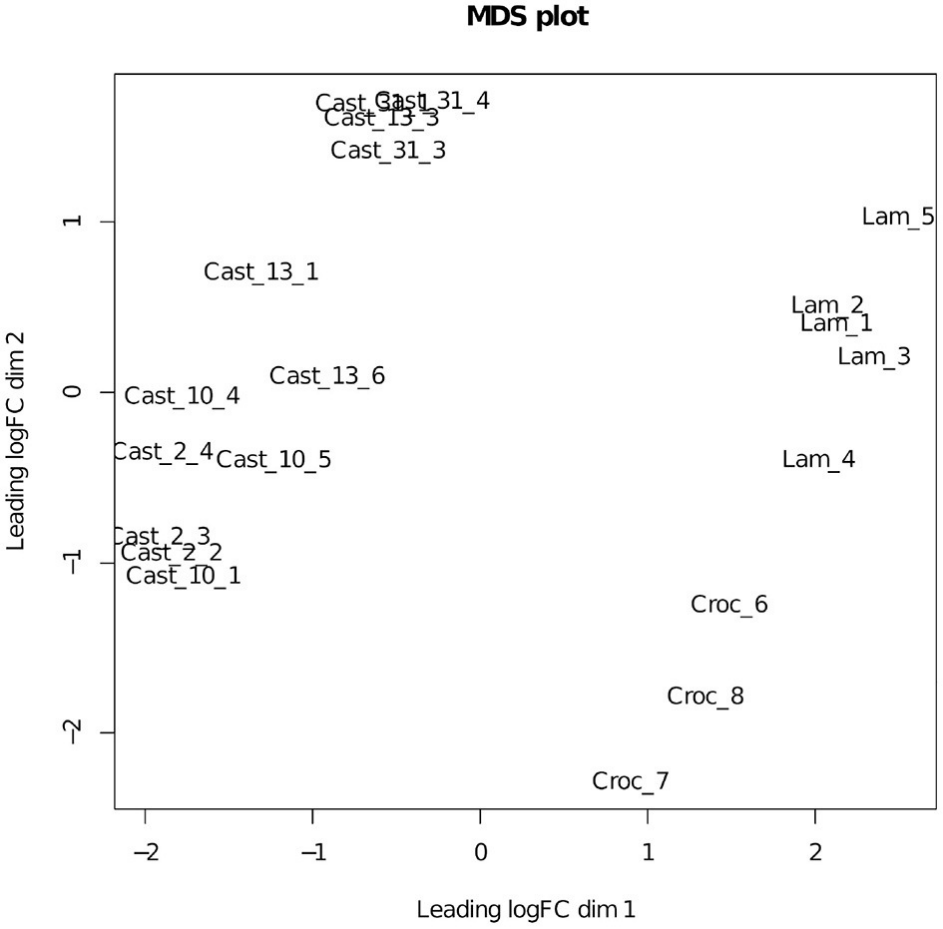
MDS plot of the additive expression matrix for *T. castellanus* and its diploid progenitors. Lam represents *T. lamottei*, Croc represents *T. crocifolius*, and Cast represents *T. castellanus*.

**Figure 3:**
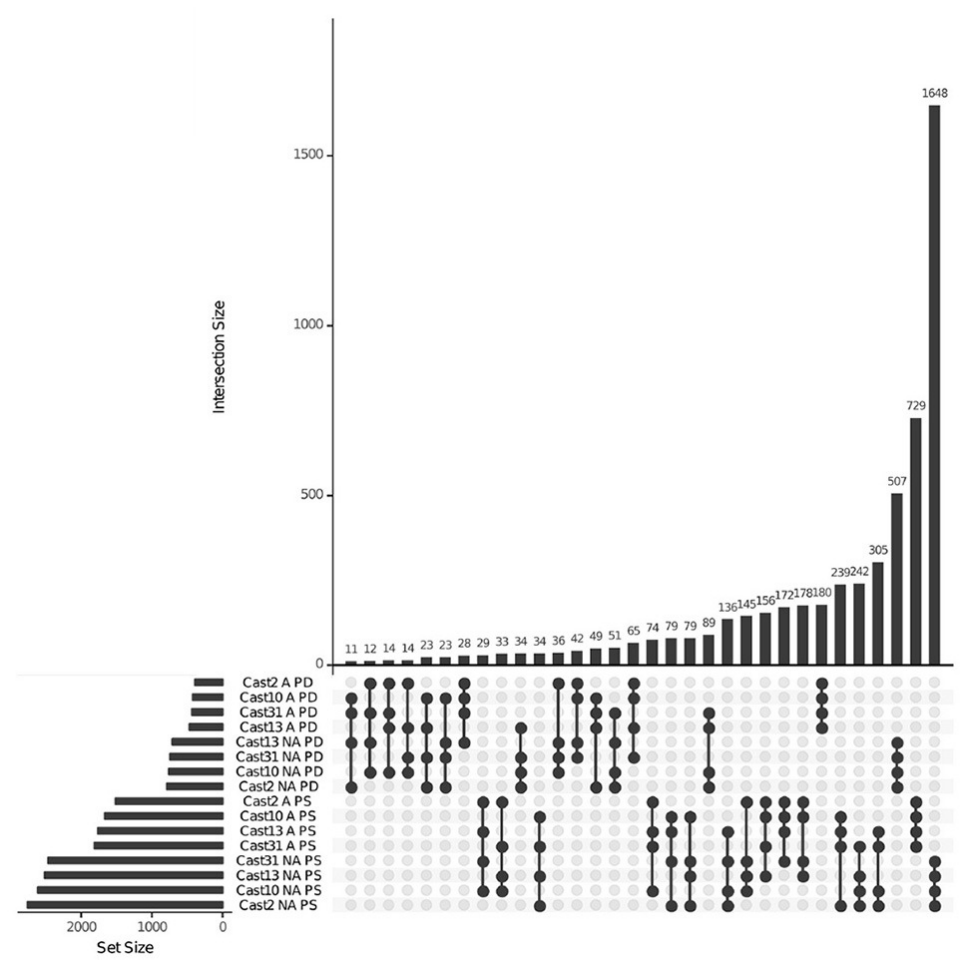
Additive and non-additive expression overlap across *T. castellanus* individuals. Set IDs represent samples that are additive (A) or non-additive (NA) where parents are different (PD) or the same (PS). Sample sets with common loci are indicated by filled circles with connecting lines, and the number of loci within that intersection may be seen directly above in the bar chart with corresponding size over each bar. The total sample sizes are found in the left bar chart and correspond to the adjacent sample.

Additivity was assessed by first performing a contrast between diploid parents to identify loci where parental expression deviated or was the same (Table 1 and Supplementary Figure 4). The matrix of counts used to estimate additive expression was subjected to multi-dimensional scaling, where samples that lie in close proximity exhibit more similar expression patterns, and plotted. The clustering of *T. castellanus* individuals is consistent with the known lineages as samples for Cast_2 and Cast_10 both come from lineage I and cluster together (Figures 1, 2, and Supplementary Figure 1). Similarly, the *T. lamottei* individuals Lam_1 and Lam_2 come from the same lineage, P-I, and are adjacent. Of the 5,806 loci remaining after filtering, parental expression was the same at 4,533 loci and different at 1,273. We found that polyploid expression is primarily non-additive where additivity was examined with respect to parental expression (Table 1), and overlap of each additive/non-additive category was assessed (Figure 3). Approximately 65% (2,155) of the loci were not additive in all four of the independent polyploids, whereas only 43% (909) of the loci consistently exhibited additivity over the four polyploids. There was no significant (FDR < 0.05) GO enrichment for shared additively expressed loci.

**Table 1:** Test for additivity in polyploid expression.

	**Not additive**	**Consistent with additive**
**Cast_2**		
Parents same	2,762 (50.6%)	1,516 (27.8%)^*^
Parents different	787 (14.4%)	391 (7.2%)^**^
**Cast_10**		
Parents same	2612 (47.9%)	1,666 (30.5%)^*^
Parents different	757 (13.9%)	421 (7.7%)^**^
**Cast_13**		
Parents same	2,515 (46.1%)	1,763 (32.3%)^*^
Parents different	710 (13.0%)	468 (8.6%)^**^
**Cast_13**		
Parents same	2,466 (45.2%)	1,812 (33.2%)^*^
Parents different	744 (13.6%)	434 (8.0%)^**^

*Counts represent loci where parental expression is not significantly different or is significantly different and polyploid expression is either additive or non-additive in the T. castellanus individuals of independent origin. Percentages are based on per-individual totals such that the expression categories of each individual sum to 100%*.

* *These loci are not strictly additive as T. castellanus expression could deviate from mid-parent expression and yet be consistent with additive when parental expression is the same*.

** *These loci have power issues because the hybrid mean expression falls within the diploid mean expression levels*.

### 3.3. Homoeolog-Specific Expression

**Figure 4:**
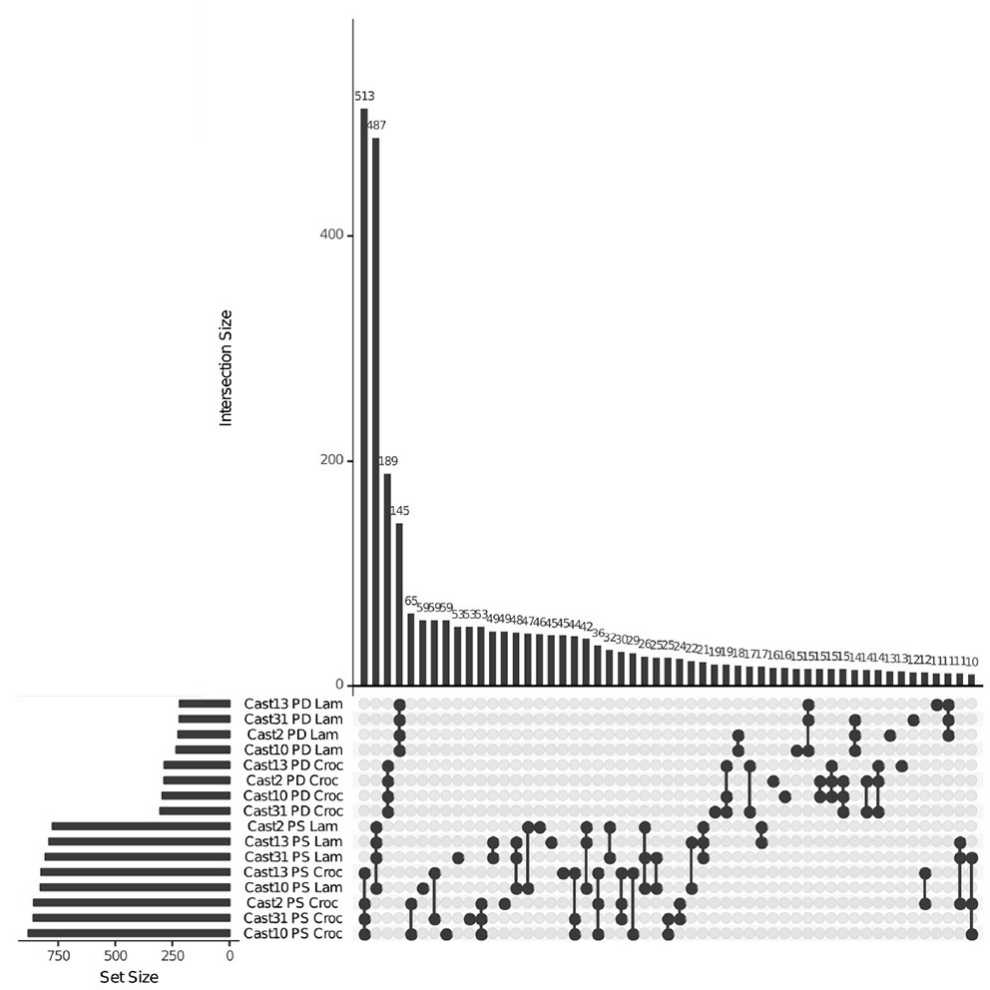
Homoeolog-specific expression overlap across *T. castellanus*. Set IDs represent samples where parents are different (PD) or the same (PS) and the direction of homoeolog-specific expression (i.e. toward higher expression of the *T. lamottei* (Lam) or *T. crocifolius* (Croc) homoeolog). Overlapping sets are indicated by filled circles, and the number of loci within that intersection may be seen directly above in the bar chart with corresponding size over each bar. The total sample sizes are found in the left bar chart and correspond to the adjacent sample.

HSE was assessed using the PG model and, similar to the additivity assessment, was examined in light of parental expression using unbiased COREs (Table 2). The number of polyploid loci exhibiting homoeolog expression bias toward each parent was similar, with a moderate, consistent bias toward *T. crocifolius* of about 50 loci, which accounts for ~7% of loci in which parental gene expression is the same but ~23% of loci which exhibit significantly non-equal expression in the parents. The percent of loci overlapping between independent polyploids was ~60% when parental expression was the same and ~64% when parental expression differed (Figure 4). There was no significant (FDR < 0.05) difference in GO enrichment for common loci demonstrating HSE.

**Table 2:** Homoeolog-Specific Expression.

	***T. lamottei***	***T. crocifolius***	**No HSE**
**(A)**			
**Parents same**			
Cast_2	774	856	1,400
Cast_10	827	881	1,346
Cast_13	789	824	1,429
Cast_31	805	858	1,397
**(B)**			
**Parents different**			
Cast_2	225	286	324
Cast_10	233	293	310
Cast_13	218	285	328
Cast_31	219	304	310
**(C)**			
**Ignoring parents**			
Cast_2	1,274	1,429	2,360
Cast_10	1,365	1,481	2,279
Cast_13	1,291	1,387	2,397
Cast_31	1,327	1,447	2,344

*Counts represent total number of loci demonstrating expression bias toward a particular parental homoeolog. Homoeolog expression biases are examined in light of **(A)** loci expression levels being the same in the diploid parents, **(B)** loci expression levels being different in the diploid parents, and **(C)** HSE if parental patterns are ignored; these numbers are higher due to filtering constraints used to determine differences in parental expression*.

### 3.4. Homoeolog Silencing and Loss

**Figure 5:**
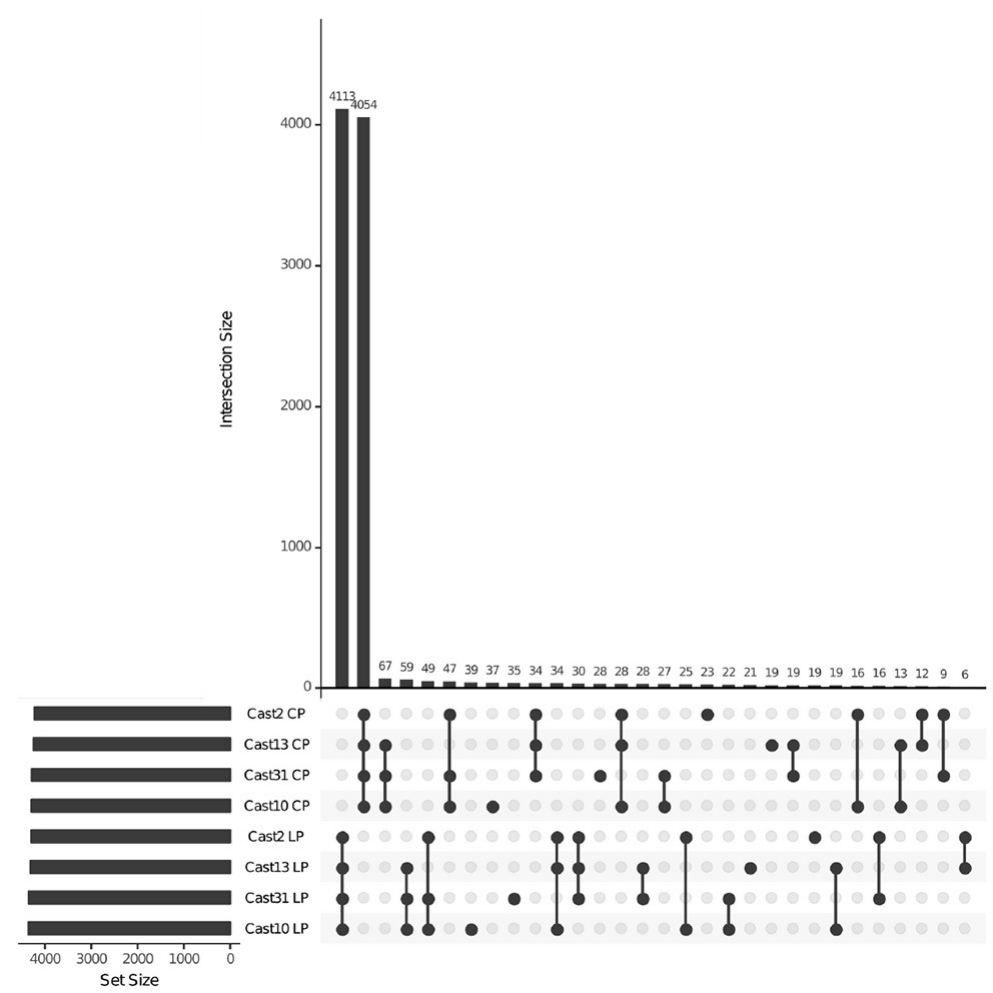
UpSet plot showing homoeologs mapping both DNA and RNA reads across *T. castellanus* individuals. Set IDs represent samples where homoeologs from *T. crocifolius* (C) or *T. lamottei* (L) are present (P) based upon both DNA and RNA alignment. Overlapping sets are indicated by filled circles, and the number of loci within that intersection may be seen directly above in the bar chart with corresponding size over each bar. The total sample sizes are found in the left bar chart and correspond to the adjacent sample. As expected, there should be no overlap across *T. crocifolius* and *T. lamottei* homoeologs.

**Figure 6:**
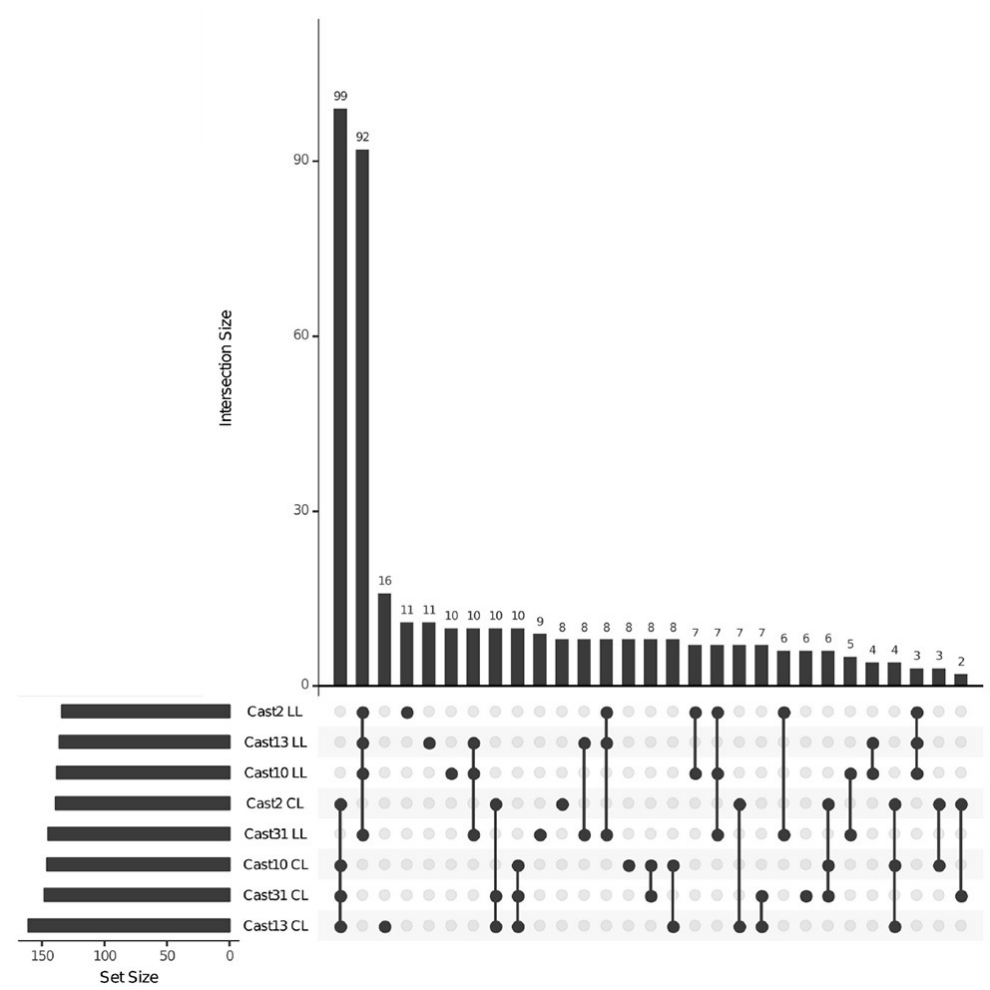
Loci lost across *T. castellanus individuals*. Set IDs represent samples where either *T. crocifolius* (C) or *T. lamottei* (L) homoeologs are lost (L). Overlapping sets are indicated by filled circles, and the number of loci within that intersection may be seen directly above in the bar chart with corresponding size over each bar. The total sample sizes are found in the left bar chart and correspond to the adjacent sample. As expected, there should be no overlap across *T. crocifolius* and *T. lamottei* homoeologs.

After orthologs were identified between diploid assemblies, exon-capture probes were designed so that genomic data could be used to distinguish between loci lost vs. silenced after polyploidization. As seen with both additivity and HSE, the number of loci expressed, silenced or lost is highly consistent across all polyploids of independent origin (Table 3). However, the degree of overlap varies among expressed, silenced or lost loci. For expressed loci, approximately 95% of the same parentally derived homoeologs (4,113 for *T. lamottei* and 4,054 for *T. crocifolius*) overlap among the four polyploids (Figure 5). Of those few homoeologs demonstrating loss, approximately 66% overlap, again, for both *T. lamottei* (92) and *T. crocifolius* (99) homoeologs, independently (Figure 6).

**Table 3:** Expression states of homoeologs derived from *T. lamottei* or *T. crocifolius*.

	***T. lamottei***				***T. crocifolius***			
	**Expressed**	**Silenced**	**Lost**	**Failed**	**Expressed**	**Silenced**	**Lost**	**Failed**
Cast_2	4,292	142	134	109	4,225	160	139	153
Cast_10	4,360	121	138	105	4,291	139	146	148
Cast_13	4,310	129	136	112	4,248	137	161	141
Cast_31	4,352	121	145	105	4,287	135	148	153

*Counts represent total number of loci exhibiting the specified expression state. T. lamottei- and T. crocifolius-derived homoeologs may be present and expressed, silenced, lost or have failed either to be isolated using sequence capture probes or spuriously mismapped reads*.

**Figure 7:**
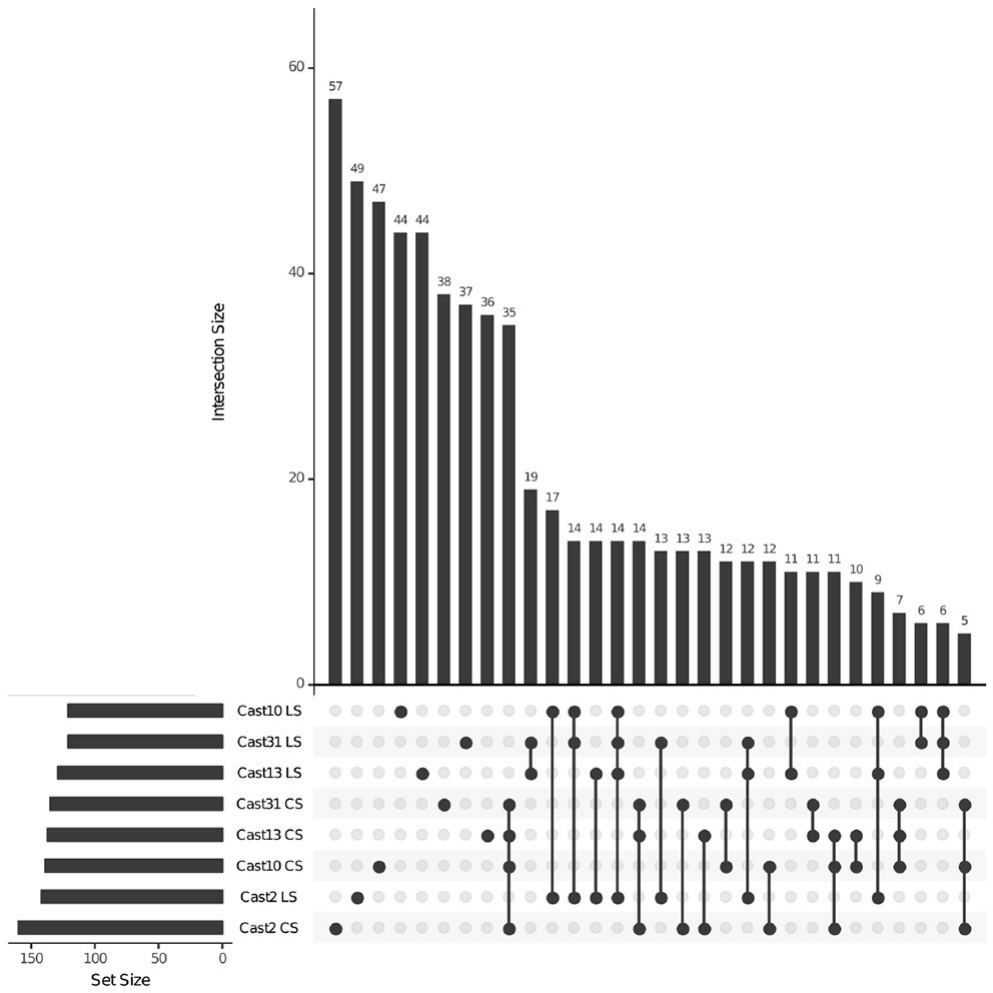
Loci silenced across *T. castellanus* individuals. Set IDs represent samples where either *T. crocifolius* (C) or *T. lamottei* (L) homoeologs are silenced (S). Overlapping sets are indicated by filled circles, and the number of loci within that intersection may be seen directly above in the bar chart with corresponding size over each bar. The total sample sizes are found in the left bar chart and correspond to the adjacent sample. As expected, there should be no overlap across *T. crocifolius* and *T. lamottei* homoeologs.

**Figure 8:**
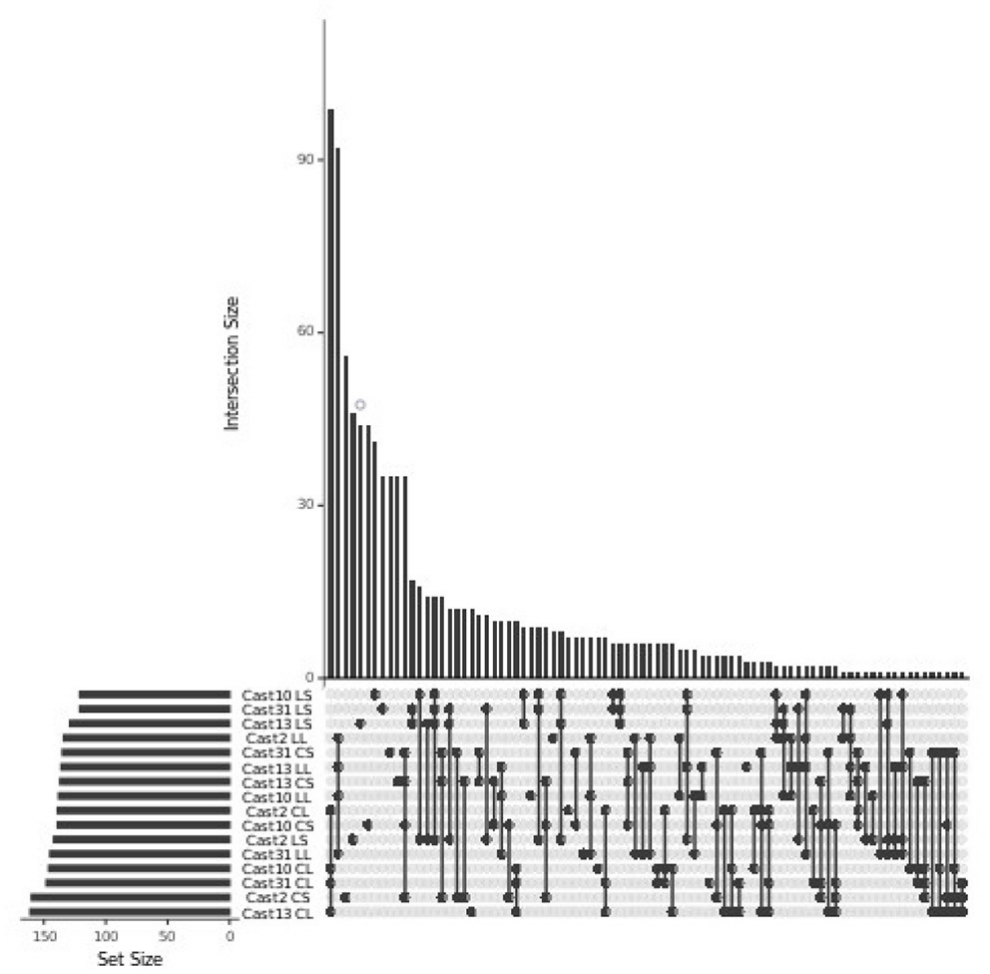
Loci lost or silenced across *T. castellanus* individuals. Set IDs represent samples where either *T. crocifolius* (C) or *T. lamottei* (L) homoeologs are lost (L) or silenced (S). Overlapping sets are indicated by filled circles, and the number of loci within that intersection may be seen directly above in the bar chart. The total sample sizes are found in the left bar chart and correspond to the adjacent sample. As expected, there should be no overlap across *T. crocifolius* and *T. lamottei* homoeologs.

Silenced homoeologs showed the most variability even though a similar number of loss events occurred across all independently formed polyploids. Only 14 *T. lamottei*-derived homoeologs (~10% of all *T. lamottei* homoeolog silencing events) and 35 *T. crocifolius*-derived homoeologs (~25% of all *T. crocifolius* homoeolog silencing events) were silenced in all four polyploids. In fact, the majority of silencing events were unique to each polyploid, suggesting that silencing is likely a much more stochastic process than homoeolog loss (Figures 7, 8). There was no significant (FDR < 0.05) GO enrichment for loci expressed, lost or silenced.

### 3.5. Functional Protein Association Network

The only group of loci that demonstrated significantly more interactions than expected based on chance included the additively expressed genes common to all independently formed allopolyploids (Supplementary Figure 5). The resulting network included 787 nodes with 185 edges with an expected edge count of 101. The average node degree was 0.47 with an average local clustering coefficient of 0.132. The FDR-corrected *q*-value was 2.75e-13.

## 4. Discussion

### 4.1. Assembly and Annotation

Transcript assembly sizes were similar in these *Tragopogon* diploids from Spain (113,865 and 155,600 contigs for *T. crocifolius* and *T. lamottei*, respectively) to those seen in the US diploid parental species (105,282, 116,777, and 122,024 for *T. dubius, T. porrifolius*, and *T. pratensis*, respectively) (Boatwright et al., 2018). The number of orthologous pairs identified between diploid progenitors was also similar, with 14,389 pairs identified in this study between *T. lamottei* and *T. crocifolius*, while US species were represented by 15,493 pairs between *T. dubius*-*T. pratensis* and 15,587 between *T. dubius*-*T. porrifolius*. Differences in CORE lengths were similar between studies with no difference over 16 bp, while the %GC difference never exceeded 5% (Supplementary Figures 2, 3). The number of hits against the SwissProt database was nearly identical for all diploid assemblies (~7,000 to *A. thaliana* and ~500 to *O. sativa* for both US and Spanish species) as were the numbers of unique eggNOG hits (~4,800). These metrics are significant in that they demonstrate that these studies contain large, similarly sized and comparable data. Thus, differences between the studies should largely be due to biological differences and not methodological differences.

### 4.2. Additive Expression

Additive and non-additive gene expression patterns are commonly studied in hybrid and polyploid plants (Guo et al., 2006; Stupar and Springer, 2006; Swanson-Wagner et al., 2006, 2009; Wang et al., 2006a,b; Baldauf et al., 2016). Synthetic *Brassica napus* exhibits proteome additivity where differential regulation was not related to protein function (Albertin et al., 2007). Additive protein expression has been previously described in the neopolyploid *Tragopogon mirus* (Koh et al., 2012), and additive gene expression in both neopolyploids *T. mirus* and *T. miscellus* (Boatwright et al., 2018).

However, expression in the diploid parents of *T. castellanus* is significantly different from that seen in the parents of the nascent polyploids. For the neopolyploids, the expression of the diploids *T. dubius* and *T. porrifolius* was the same for 5,806 loci and different for 4,706 loci; *T. dubius* and *T. pratensis* expression was the same for 5,026 and different for 5,121 loci (Boatwright et al., 2018). While expression was different between diploid parents for the neopolyploids about 50% of the time, the values seen here for *T. crocifolius* and *T. lamottei* indicate that parental expression is primarily the same. Similarly, whereas the homoeolog expression was consistent with additivity for the majority of loci within the neopolyploids (Boatwright et al., 2018), plants of the mesopolyploid *T. castellanus* exhibit more non-additive expression.

A recent publication in *Spartina* (Giraud et al., 2021) has demonstrated that a high degree transcriptome repatterning (52% of genes deviated from parental additivity) occurs following neopolyploidy (within the last 170 years), and long-term, divergent transcriptome evolution is evident between the mesohexaploid parents that diverged 2-3 MYA (with 36% genes deviating from parental additivity).

One potential reason for this difference in additive expression may be that neopolyploid survival is dependent upon reduction in gene expression noise, as expression noise can have negative impacts upon fitness (Barkai and Leibler, 2000; Rao et al., 2002; Fraser et al., 2004; Pires and Conant, 2016). Thus, shrinkage toward mean parental expression within neopolyploids may alleviate the effects of transcriptomic shock, especially for polyploids with sub-genome *trans*-acting factors that are largely interchangeable. Over longer periods of time, mutation and selection may then optimize expression of genes, resulting in more non-additive expression. Both noise reduction and gene dosage are expected to play a large role after polyploidization (reviewed in Conant et al., 2014; Pires and Conant, 2016). Interestingly, dosage effects are seen in numerous additively expressed genes within polyploids (Guo et al., 1996; Chen, 2007). These dosage effects are expected to primarily affect genes that function in protein complexes or biological pathways (reviewed in Freeling, 2009; Birchler and Veitia, 2010, 2012). This explanation appears to be the case for additively expressed genes conserved across these independently formed polyploids in that our functional protein association network was significantly enriched for protein-protein interactions.

There is evidence that members of protein complexes within yeast, fruit flies, and humans all exhibit reduced expression noise (Ohno, 1970; Lemos et al., 2004; Schuster-Böckler et al., 2010; reviewed in Pires and Conant, 2016). As such, finding protein-protein interaction enrichment among additively expressed genes may be further evidence that noise reduction and dosage play a significant role in expression changes after allopolyploidization. The degree of dissimilarity between parental expression may also significantly affect homoeolog expression fate between neo- and mesopolyploids (Conant et al., 2014). Environmental differences may also select for different expression patterns over time (Otto and Whitton, 2000). As such, there is likely a complex interplay among the processes governing expression patterns after polyploidization.

### 4.3. Homoeolog-Specific Expression

HSE, also sometimes called homoeolog expression bias, has been observed in neopolyploids such as *Senecio* (Hegarty et al., 2012), mesopolyploids such as *Gossypium* (Adams et al., 2004; Chaudhary et al., 2009; Yoo et al., 2013), and even more broadly across polyploid plants (Buggs et al., 2010b; Schnable et al., 2011; Grover et al., 2012; Woodhouse et al., 2014; Yang et al., 2016). Notably, we observed numerous loci demonstrating HSE, but overall, we see a similar proportion of loci exhibiting homoeolog expression bias toward each parent (Grover et al., 2012). This balanced proportion of HSE in *Tragopogon* is interesting in that numerous other allopolyploid plants have exhibited substantial subgenome expression bias (Chen and Pikaard, 1997; Wang et al., 2006b; Flagel et al., 2008; Chaudhary et al., 2009; Akhunova et al., 2010; Schnable and Freeling, 2011; Schnable et al., 2011). However, both neoallopolyploid *Tragopogon* (Boatwright et al., 2018) and the mesoallopolyploid *T. castellanus* demonstrate similar proportions of homoeolog expression bias toward their corresponding parents. HSE in resynthesized *Brassica* neoallopolyploids is established soon after the initial genome merger and allopolyploidization (Yang et al., 2016). So, HSE is potentially yet another ameliorative response to whole-genome duplication and/or hybridization (Pires and Conant, 2016). The cause of these expression patterns in *Tragopogon* is unclear, but numerous genetic and epigenetic mechanisms have been proposed to affect expression in polyploids (Chen, 2007).

The maintenance of dosage balance is not likely to occur indefinitely after whole-genome duplication (Conant et al., 2014; McGrath et al., 2014). HSE is believed to allow duplicated copies to undergo subfunctionalization, neofunctionalization or fractionation, but it is possible that recurrent gene conversion between duplicated copies may maintain sequence identity between them (Pires and Conant, 2016). Biased sub-genome expression dominance has been observed following whole-genome duplication in maize where biased expression occurs within neofunctionalized regulatory genes, and non-regulatory neofunctionalized genes incrementally acquire sub-genome dominance during development (Hughes et al., 2014). Epigenetic regulation has been shown to facilitate sub-genome dominance after whole-genome triplication in *B. rapa* where a biased distribution of transposable elements among sub-genomes as well as small targeting RNAs are responsible for expression dominance at a sub-genome scale (Cheng et al., 2016). It is also possible that HSE reconciles problems arising from heterologous protein complexes for proteins that function more efficiently as homopolymers or require precise binding affinities, stoichiometry or product ratios (Birchler and Veitia, 2010, 2012; Boatwright et al., 2018).

### 4.4. Homoeolog Silencing and Loss

The process of genome evolution after polyploidization is characterized by alterations in methylation, transposable element activity, expression and function changes as well as genome rearrangement and downsizing (reviewed in Van de Peer et al., 2009; Wendel, 2015; Soltis et al., 2016; Wendel et al., 2018). While these changes have been observed in mesopolyploids (Wang et al., 2011) and paleopolyploids (Schnable et al., 2011), they also occur in neopolyploids where a wide spectrum of genomic changes may occur soon after genome merger and duplication (Madlung and Wendel, 2013), indicating that neopolyploid genomes are not necessarily additive or static (Leitch et al., 2008). Stochastic silencing has been proposed to play an important role in the formation of new species and diploidization after polyploidization. Polyploid species are notable in their tendency to preserve duplicate gene copies, which could be a result of gene dosage effects (Lynch and Conery, 2000; Conant et al., 2014). Dynamic silencing likely serves as a damage-control mechanism to temper potentially adverse effects of polyploidization on gene dosage to improve chances of establishment and adaptation of nascent polyploids (Wendel, 2000; Chaudhary et al., 2009; Buggs et al., 2011). In this study, the silencing of specific homoeologs was more inconsistent across independent polyploids than were loss events or expressed genes. In fact, the majority of silencing events were unique to each polyploid, which seems to support the role of stochastic silencing in polyploid plants. However, it is notable that even though silencing appeared to be stochastic, the homoeologs that were lost were more consistent. This may suggest that the mechanisms governing fractionation are more systematic.

*Tragopogon castellanus* was previously shown to exhibit a nearly additive genome size of its parents, and the degree of loss seen here (~3% of loci examined) is consistent with that finding (Mavrodiev et al., 2015). Neopolyploid *Tragopogon* species from the US also exhibited very little putative gene loss (Boatwright et al., 2018) and exhibit an additive genome size (Pires et al., 2004a). Long-term gene loss and retention after whole-genome duplication has demonstrated what appears to be a non-random progression in previous studies (Barker et al., 2008; Freeling, 2009; Birchler and Veitia, 2010; Schnable et al., 2011; Severin et al., 2011; De Smet et al., 2013; Soltis et al., 2016). These observations may also be consistent with the biased fractionation hypothesis, where genome dominance is expected when the subgenomes are highly diverged but not when the subgenomes are similar (Garsmeur et al., 2014; Zhao et al., 2017). While the exact divergence between for diploid parents of *T. castellanus* has not been thoroughly investigated, the P-derived parental genetic divergence index, the ratio between parental divergence and the average genetic divergence in the respective genus, is 1.14 (Paun et al., 2009), indicating that the balanced expression may be justified by the low parental divergence. This biased fractionation theory is also supported by the contrasting case of recently formed *Mimulus peregrinus* allopolyploids (Edger et al., 2017) where subgenome expression dominance occurs immediately following the hybridization of divergent genomes and increases significantly over subsequent generations and results from *Ephedra* allotetraploids whose subgenomes are approximately 8MY diverged, where it has been shown that the rapid formation of large genomes could be attributed to even and slow fractionation following polyploidization (Wu et al., 2021).

*Tragopogon* seems to be yet another case of convergent homoeolog loss after multiple, independent polyploidization events similar to recent results from *Capsella* allotetraploids have demonstrated predictable patterns of gene retention and loss following polyploidization (Douglas et al., 2015). We further checked for gene ontology enrichment within our retained, lost and silenced genes but found no significant enrichment. Differential regulation of proteome additivity was not related to protein function in *Brassica napus* allotetraploids (Albertin et al., 2007). So, a lack of enrichment within additively expressed genes may be expected. While the lack of enrichment within lost genes contrasts with studies that found binding proteins, protein kinases, transcription factors, and transferases are usually retained in duplicate (Jiao et al., 2011), and photosynthesis and cell cycle genes typically drop to singleton status (De Smet et al., 2013), it is the same result found in the neopolyploid *Tragopogon* species (Boatwright et al., 2018). It may be that loss is not predominantly determined by functional category but rather by some other genetic or epigenetic characteristic such as noise reduction or dosage, at least within *Tragopogon*.

## 5. Final Remarks

The short- and long-term effects of *cis*- and *trans*-acting interactions are sure to have a significant, if not dynamically different, effect on duplicate gene fate within allopolyploid species. Studies of these processes lack duplication but are certain to identify broader physiological, ecological, and evolutionary implications of polyploidization (Soltis et al., 2016). Here, we compared both homoeolog fate convergence within independently formed mesoallopolyploid populations (*T. castellanus*) and how those compare to neoallopolyploids within the same genus using the same methodology. While homoeolog expression bias was balanced in both the two neopolyploids and in the mesopolyploid, the degree of additive expression was significantly different, with populations of the mesopolyploid demonstrating more non-additive expression. We found that homoeologs that are retained or lost seem to be strongly convergent across independently formed allopolyploids, while silencing tends to occur stochastically. Further, this non-random trend in long-term homoeolog retention and loss is not unique to *Tragopogon* but may be selectively advantageous for polyploid speciation and survival (Barker et al., 2008; Freeling, 2009; Birchler and Veitia, 2010; Severin et al., 2011; Schnable et al., 2012; De Smet et al., 2013; Soltis et al., 2016). While there was no GO enrichment among the studied gene sets, additively expressed genes demonstrated enrichment for protein-protein interactions within a functional network. It may be that gene dosage and noise minimization play leading roles in regulating gene expression patterns after allopolyploidization, and these patterns are conserved across independent lineages.

## Data Availability Statement

The data presented in this study are deposited in the NCBI's Sequence Read Archive (SRA) under BioProject PRJNA728143. Scripts used to run the analyses are available on GitHub at https://github.com/jlboat/Tragopogon_castellanus.

## Author Contributions

JB, DS, PSo, PSc, and WB designed the experiments. H-CH and AS generated data. JB and C-TY performed analyses. JB, WB, PSo, and DS wrote the manuscript. All authors contributed to the article and approved the submitted version.

## Conflict of Interest

PSc is a Changjiang Scholar at China Agriculture University, and co-founder and managing partner of Data2Bio, LLC; Dryland Genetics, LLC; and EnGeniousAg, LLC. He is a member of the scientific advisory board and a shareholder of Hi-Fidelity Genetics, Inc. and a member of the scientific advisory boards of Kemin Industries and Centro de Tecnologia Canavieira. H-CH was employed by the company Covance Inc. The remaining authors declare that the research was conducted in the absence of any commercial or financial relationships that could be construed as a potential conflict of interest.
