# Supplementary material for: Trajectories of Homoeolog-Specific Expression in Allotetraploid Tragopogon castellanus Populations of Independent Origins
Source: Front Plant Sci. 2021 Jun 23;12:679047. doi: 10.3389/fpls.2021.679047 (PMC8261302; doi:10.3389/fpls.2021.679047)
Supplement: Supplementary file 1 [file Data_Sheet_1.DOCX]

Supplementary Table 1. Sample information

| Sample ID | Pop-Ind | Species | Lineage | Parent Accession No. |
| --- | --- | --- | --- | --- |
| 1 | 18-2 | *T. lamottei* | P-I | 3576 |
| 2 | 25-6 | *T. lamottei* | P-I | 3582 |
| 3 | 9-2 | *T. lamottei* | P-II | 3569 |
| 4 | 9-17 | *T. lamottei* | P-II | 3630 |
| 5 | 9-18 | *T. lamottei* | P-II | 3629 |
| - | - | *-* | - | - |
| 6 | A-5 | *T. crocifolius* | P-B | 3609 |
| 7 | A-3 | *T. crocifolius* | P-B | 3607 |
| 8 | B-4 | *T. crocifolius* | P-B | 3611 |
| - | - | *-* | - | - |
| Cast2-2-1 | 2-2-1 | *T. castellanus* | I | - |
| Cast2-3-4 | 2-3-4 | *T. castellanus* | I | - |
| Cast2-4-2 | 2-4-2 | *T. castellanus* | I | - |
| Cast10-1-4 | 10-1-4 | *T. castellanus* | I | - |
| Cast10-4-4 | 10-4-4 | *T. castellanus* | I | - |
| Cast10-5-3 | 10-5-3 | *T. castellanus* | I | - |
| Cast13-1-1 | 13-1-1 | *T. castellanus* | IIa | - |
| Cast13-3-5 | 13-3-5 | *T. castellanus* | IIa | - |
| Cast13-6-4 | 13-6-4 | *T. castellanus* | IIa | - |
| Cast31-1-3 | 31-1-3 | *T. castellanus* | IIb | - |
| Cast31-3-4 | 31-3-4 | *T. castellanus* | IIb | - |
| Cast31-4-1 | 31-4-1 | *T. castellanus* | IIb | - |

Supplementary Table 1 cont.

| Population | Longitude | Latitude | Location |
| --- | --- | --- | --- |
| 3040 | 42.7237972 | -0.2995639 | Slope, meadow habitat. Road to Panticosa |
| 3049 | 42.5077528 | -0.6674944 | Hill overlooking Torla. |
| 3031 | 42.883588 | -4.491234 | Road out of Cervera de Pisuerga |
| 3031 | 42.883588 | -4.491234 | Road out of Cervera de Pisuerga |
| 3031 | 42.883588 | -4.491234 | Road out of Cervera de Pisuerga |
| - | - | - | - |
| A (Sicily) | 37.8787 | 14.017869 | Site A: Sicily, Madonie Mts., mountsides of Carbonara Mts., next to Piano Battaglia |
| A (Sicily) | 37.8787 | 14.017869 | Site A: Sicily, Madonie Mts., mountsides of Carbonara Mts., next to Piano Battaglia |
| A (Sicily) | 37.8787 | 14.017869 | Site B: Sicily, Madonie Mts., mountsides of Carbonara Mts., next to Piano Battaglia |
| - | - | - | - |
| 3024 | 41.055914 | -2.641785 | Edge of town, Siguenza |
| 3024 | 41.055914 | -2.641785 | Edge of town, Siguenza |
| 3024 | 41.055914 | -2.641785 | Edge of town, Siguenza |
| 3032 | 42.887935 | -4.487954 | At scenic overlook |
| 3032 | 42.887935 | -4.487954 | At scenic overlook |
| 3032 | 42.887935 | -4.487954 | At scenic overlook |
| 3035 | 42.507753 | -0.6674944 | Monastery Neuvo S. Juan de la Pena |
| 3035 | 42.507753 | -0.6674944 | Monastery Neuvo S. Juan de la Pena |
| 3035 | 42.507753 | -0.6674944 | Monastery Neuvo S. Juan de la Pena |
| 3055 | 42.490833 | -0.0997222 | Highway N-260 km 462, Arresa |
| 3055 | 42.490833 | -0.0997222 | Highway N-260 km 462, Arresa |
| 3055 | 42.490833 | -0.0997222 | Highway N-260 km 462, Arresa |


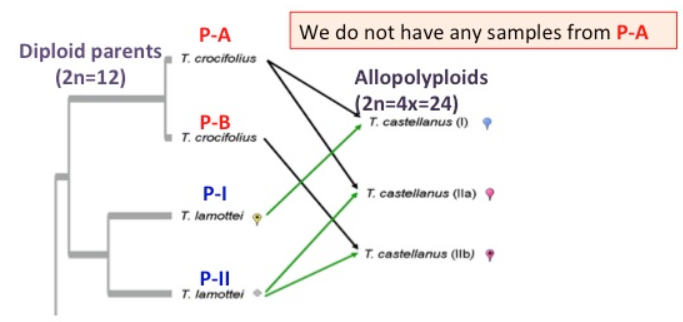


Supplementary Figure 1. Relationship among the diploid accessions and independently formed allopolyploids.

Supplementary Table 2. RNA read counts after trimming

|  | Sample Num | R1 reads | R2 reads | Merged reads |
| --- | --- | --- | --- | --- |
| *T. lamottei* | 1 | 8,866,088 | 8,866,088 | 7,392,889 |
| *T. lamottei* | 2 | 8,399,419 | 8,399,419 | 7,189,102 |
| *T. lamottei* | 3 | 7,373,019 | 7,373,019 | 6,397,006 |
| *T. lamottei* | 4 | 8,453,785 | 8,453,785 | 8,047,433 |
| *T. lamottei* | 5 | 9,678,180 | 9,678,180 | 7,781,350 |
| *T. crocifolius* | 6 | 8,932,807 | 8,932,807 | 6,806,793 |
| *T. crocifolius* | 7 | 8,599,325 | 8,599,325 | 8,100,111 |
| *T. crocifolius* | 8 | 8,960,760 | 8,960,760 | 7,803,074 |

Supplementary Table 3. DNA read counts after trimming

|  | Sample ID | R1 reads | R2 reads | Unpaired reads |
| --- | --- | --- | --- | --- |
| T. lamottei | 9-18-1 | 68844941 | 68844941 | 2680103 |
| T. lamottei | 25-6B-1 | 46771892 | 46771892 | 2338190 |
| T. lamottei | 18-5D-1 | 24594180 | 24594180 | 770016 |
| T. crocifolius | B-4-1 | 56797189 | 56797189 | 2263364 |
| T. castellanus | 10-1-4 | 57260609 | 57260609 | 2470051 |
| T. castellanus | 10-4-4 | 73132096 | 73132096 | 2848761 |
| T. castellanus | 10-5-3 | 74963734 | 74963734 | 3031331 |
| T. castellanus | 13-1-1 | 23909923 | 23909923 | 809532 |
| T. castellanus | 13-3-5 | 51329417 | 51329417 | 2189769 |
| T. castellanus | 13-6-4 | 20800537 | 20800537 | 835156 |
| T. castellanus | 2-2-1 | 48906711 | 48906711 | 2344188 |
| T. castellanus | 2-3-5 | 55020716 | 55020716 | 2325974 |
| T. castellanus | 2-4-2 | 49004615 | 49004615 | 2112721 |
| T. castellanus | 31-1-3 | 105047132 | 105047132 | 5166823 |
| T. castellanus | 31-3-4 | 13769836 | 13769836 | 629169 |
| T. castellanus | 31-4-1 | 103467457 | 103467457 | 5092982 |

Supplementary Table 4. Program Versions and Parameters

|  | Program | Version |  |
| --- | --- | --- | --- |
|  | bedtools | 2.24.0 |  |
|  | Biopython | 1.65 |  |
|  | Bowtie | [v0.12.9, -m 1, -v 3] |  |
|  | CAP3 | 2012-07-05 |  |
|  | Cutadapt | [v1.7.1, -O=5] |  |
|  | HMMER | 3.1b1 |  |
|  | Last | [v531, -l 25] |  |
|  | MAFFT | [v7.127] |  |
|  | NCBI BLAST | 2.2.29 |  |
|  | Rnammer | 1.2 |  |
|  | SignalP | 4.1c |  |
|  | tmhmm | 2.0c |  |
|  | Trimmomatic | [v0.32] |  |
|  | Trinity | [v2013-02-25] |  |
|  | Trinotate | 3.0.1 |  |
|  | WU-BLAST | 2.0 |  |

Supplementary Table 5. R session information

| PG model | R version 3.0.2 (2013-09-25) | | |  |  |  |
| --- | --- | --- | --- | --- | --- | --- |
|  | Platform: x86_64-unknown-linux-gnu (64-bit) | | | |  |  |
|  |  |  |  |  |  |  |
|  | locale: |  |  |  |  |  |
|  | [1] LC_CTYPE=en_US.UTF-8 LC_NUMERIC=C | | | | |  |
|  | [3] LC_TIME=en_US.UTF-8 LC_COLLATE=en_US.UTF-8 | | | | |  |
|  | [5] LC_MONETARY=en_US.UTF-8 LC_MESSAGES=en_US.UTF-8 | | | | | |
|  | [7] LC_PAPER=en_US.UTF-8 LC_NAME=C | | | | |  |
|  | [9] LC_ADDRESS=C LC_TELEPHONE=C | | | | |  |
|  | [11] LC_MEASUREMENT=en_US.UTF-8 LC_IDENTIFICATION=C | | | | | |
|  |  |  |  |  |  |  |
|  | attached base packages: | |  |  |  |  |
|  | [1] stats graphics grDevices utils datasets methods base | | | | | |
|  |  |  |  |  |  |  |
|  | loaded via a namespace (and not attached): | | | |  |  |
|  | [1] tools_3.0.2 | |  |  |  |  |
|  |  |  |  |  |  |  |
| voom | R version 3.2.0 (2015-04-16) | | |  |  |  |
|  | Platform: x86_64-unknown-linux-gnu (64-bit) | | | |  |  |
|  | Running under: Red Hat Enterprise Linux Server release 6.8 (Santiago) | | | | | |
|  |  |  |  |  |  |  |
|  | locale: |  |  |  |  |  |
|  | [1] LC_CTYPE=en_US.UTF-8 LC_NUMERIC=C | | | | |  |
|  | [3] LC_TIME=en_US.UTF-8 LC_COLLATE=en_US.UTF-8 | | | | |  |
|  | [5] LC_MONETARY=en_US.UTF-8 LC_MESSAGES=en_US.UTF-8 | | | | | |
|  | [7] LC_PAPER=en_US.UTF-8 LC_NAME=C | | | | |  |
|  | [9] LC_ADDRESS=C LC_TELEPHONE=C | | | | |  |
|  | [11] LC_MEASUREMENT=en_US.UTF-8 LC_IDENTIFICATION=C | | | | | |
|  |  |  |  |  |  |  |
|  | attached base packages: | |  |  |  |  |
|  | [1] stats graphics grDevices utils datasets methods base | | | | | |
|  |  |  |  |  |  |  |
|  | other attached packages: | |  |  |  |  |
|  | [1] edgeR_3.12.1 limma_3.26.9 | | |  |  |  |

Supplementary Figure 2. *T. lamottei* and *T. crocifolius* CORE lengths mean-difference plot. Mean-difference plot demonstrating that differences in CORE lengths never exceed 16 bp. Length difference is calculated as *T. lamottei* – *T. crocifolius* length.

Supplementary Figure 3. *T. lamottei* and *T. crocifolius* CORE Percent GC mean-difference plot. Mean-difference plot demonstrating that differences in CORE percent GC content never exceeded 5%. Percent GC difference is calculated as *T. lamottei* – *T. crocifolius* percent GC content.

Supplementary Figure 4. Voom mean-variance trend using the additive expression matrix for *T. castellanus* and its diploid progenitors.


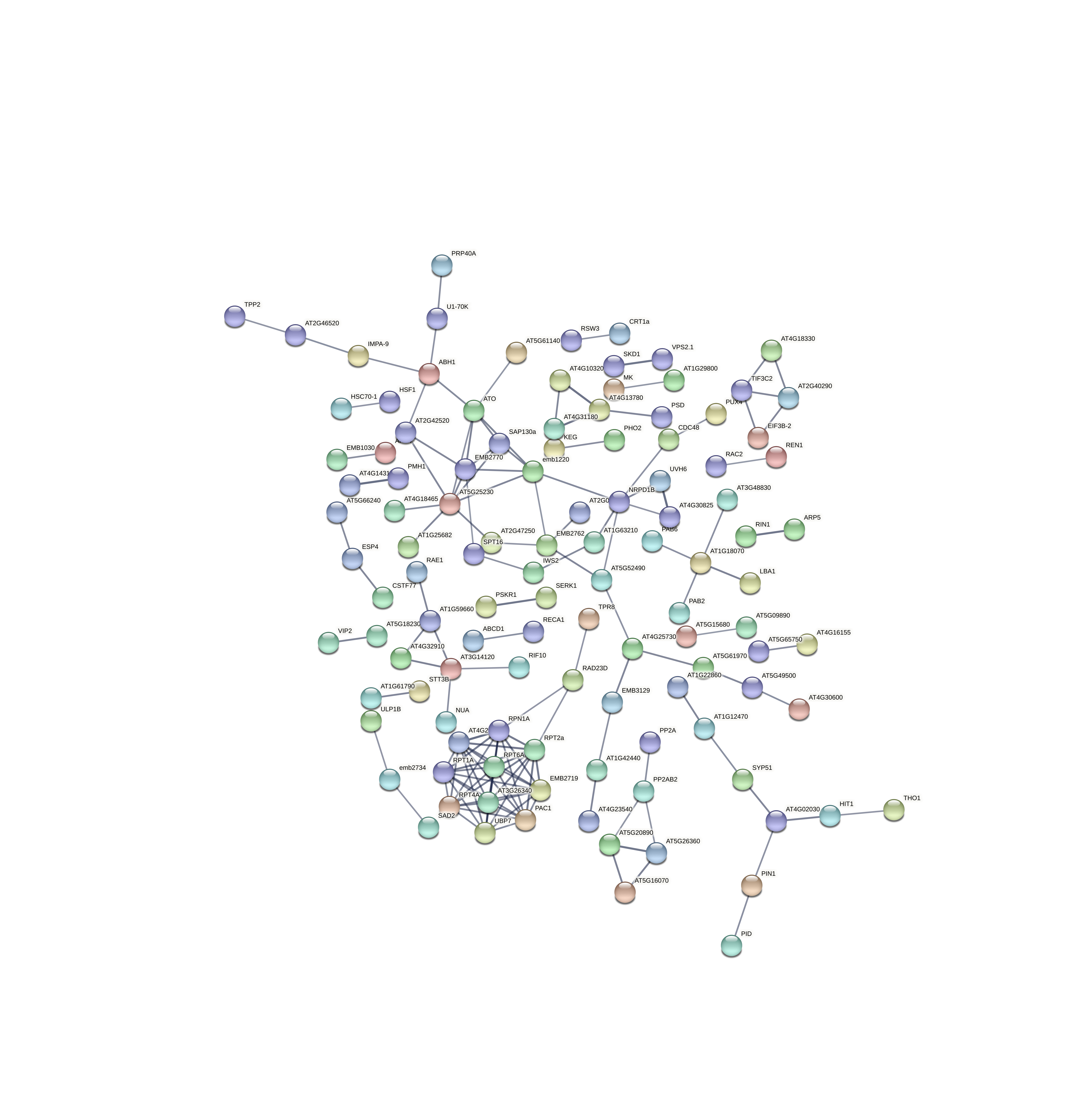


Supplementary Figure 5. Functional protein association network of additively expressed genes common to all polyploid individuals.
